# On the Compliance of Women Engineers with a Gendered Scientific System

**DOI:** 10.1371/journal.pone.0145931

**Published:** 2015-12-30

**Authors:** Gita Ghiasi, Vincent Larivière, Cassidy R. Sugimoto

**Affiliations:** 1 Department of Mechanical and Industrial Engineering, Concordia University, 1455 De Maisonneuve Blvd. W., Montréal, QC, H3G 1M8, Canada; 2 École de bibliothéconomie et des sciences de l’information, Université de Montréal, C.P. 6128, Succ. Centre-Ville, Montréal, QC, H3C 3J7, Canada; 3 Observatoire des Sciences et des Technologies (OST), Centre Interuniversitaire de Recherche sur la Science et la Technologie (CIRST), Université du Québec à Montréal, CP 8888, Succ. Centre-Ville, Montréal, QC, H3C 3P8, Canada; 4 School of Informatics and Computing, Indiana University, 1320 E. 10th St, Bloomington, IN, 47405, United States of America; Katholieke Universiteit Leuven, BELGIUM

## Abstract

There has been considerable effort in the last decade to increase the participation of women in engineering through various policies. However, there has been little empirical research on gender disparities in engineering which help underpin the effective preparation, co-ordination, and implementation of the science and technology (S&T) policies. This article aims to present a comprehensive gendered analysis of engineering publications across different specialties and provide a cross-gender analysis of research output and scientific impact of engineering researchers in academic, governmental, and industrial sectors. For this purpose, 679,338 engineering articles published from 2008 to 2013 are extracted from the Web of Science database and 974,837 authorships are analyzed. The structures of co-authorship collaboration networks in different engineering disciplines are examined, highlighting the role of female scientists in the diffusion of knowledge. The findings reveal that men dominate 80% of all the scientific production in engineering. Women engineers publish their papers in journals with higher Impact Factors than their male peers, but their work receives lower recognition (fewer citations) from the scientific community. Engineers—regardless of their gender—contribute to the reproduction of the male-dominated scientific structures through forming and repeating their collaborations predominantly with men. The results of this study call for integration of data driven gender-related policies in existing S&T discourse.

## Introduction

Innovation—and the subsequent enhancement of knowledge-based economies [1,2]—has been identified as a critical catalyst for social development, economic growth, and prosperity [3–5]. It has been argued that the pervasive gender inequalities in science [6] inhibit optimization of these knowledge-based economies [7]. Furthermore, correlates have been identified between gender-inequalities in scientific production and other economic variables (e.g., gross national income) [8]. Therefore, the construction of science, technology, and innovation (STI) policies must take into account the economic, social, and cultural factors that constrain women from engaging in scientific activities and perpetuate gender inequality [9].

Metaphors and catch phrases abound in describing the underrepresentation of women in science: the “productivity puzzle” questions the lower rate of female production of scientific works [10], the “leaky pipeline” [11] explains women’s attrition in scientific work, and the “glass ceiling” speaks to the lack of advancement [12–14]. Women are said to toil under a “triple burden” of gender stereotypes, professional obligations, and the stress inherent in dealing with lack of social capital and limited opportunities and respond to the environment in four ways: (1) adapt to the current scientific system; (2) search for other science-related careers; (3) leave science; or (4) change the structure of science [15]. The last is perhaps the least common response and has been unsuccessful in many ways. Despite numerous initiatives to eradicate disparities, the pipeline still leaks women at a rate similar to the rates in the 1970s [16] and few women are in the highest echelons of science [17].

Cole and Zuckerman’s [10] question of the productivity puzzle fueled academic interest in studying the persistence of gender disparity in research productivity (operationalized as number of publications) [6,18–24]. Impact—measured by number of citations received—has also dominated scientometric studies of gender disparities, with differing results by discipline and country [6,21,25–30], demonstrating the need for discipline-focused studies of gender disparities.

Collaboration is one factor that has been shown to be positively related to increased productivity for female scientists [25,31,32]. Recent research has shown that female scientists collaborate—that is, co-author scientific papers—proportionally more than their male counterparts [33,34], contradicting earlier research on the topic [23,31]. This propensity for collaboration may have positive impact on the production of new knowledge: gender diverse teams have also been shown to lead to increased team success [35,36]. However, the type of collaborations in which women engage is not equal—for example, female scientists are also more likely to engage in domestic collaborations, rather than in international collaborations, which has severe implications for citation impact [6,21].

Social network analysis (SNA) has emerged as a useful method for identifying the structure of collaboration networks in science. Newman [37] examined the density of collaboration networks across fields, finding significant differences between theoretical and experimental domains. These differences have led to several discipline-specific studies (e.g., [38]). Studies have also been conducted to investigate the evolution and statistical properties of co-authorship networks (e.g., [39–42]). However, few studies have isolated the role of women in scientific collaboration networks. Exceptions include Whittington's [43] analysis of the co-inventorship network of life scientists in the Boston region, Badar’s [44] analysis of chemistry researchers in Pakistan (which showed a stronger positive relationship of degree and closeness centrality for women), and the work of Ozel et al. [34], which demonstrated that women are more likely to co-author with their female counterparts.

Contextual factors are often overlooked in studies of productivity and impact—that is, the context in which the authors work (e.g., academic, corporate, government) are rarely included in the analyses. In the area of patent analysis, studies have suggested gender differences may be a direct result of the environments in which the work is conducted [43,45,46]. Policies, therefore, should be contextualized by knowledge of the social, cultural, and organizational factors that influence women’s retention and success in science [12,14].

Engineering is typically associated with technological advancements and innovation, which is central to many countries’ economic growth [47]. Yet engineering fails to meet its promises for societal development, as women are largely underrepresented in this discipline [48–50]. Attempts for integration of more women in engineering are proven to be only partially successful, as gender disparities are also rooted in cultural associations among engineering, technology, and masculinity [14,50]. As a consequence, women are likely to face greater difficulties coping with the engineering culture [51], which Faulkner [52] referred to it as ‘in/visibility paradox’, as women are often visible as women but invisible as engineers, and they constantly have to work harder to prove themselves as professionals. In the growing literature and focus on the capacity building for women in science, technology, engineering, and mathematics (STEM), there is a lack of focus on women’s scientific participation and performance in engineering specialties. There remains, therefore, a clear need for disciplinarily homogeneous, contextually enhanced, and methodologically rigorous studies of gender disparities in engineering. This paper seeks to address this void and answers the following questions: 1) what is the gender profile of the engineering authorship? and 2) what is the role of female authors in the diffusion of engineering knowledge?

For this purpose, the present study evaluates gender differences in contemporary (i.e., 2008–2013) scientific production and impact of engineers, by sector of affiliation (i.e., academic, governmental, industrial). It further scrutinizes the engineering scientific community and analyzes cross-gender co-authorship collaboration patterns among researchers, mapping the position of female and male engineers in a complex network of scientific collaborations.

## Methods

### Data

Bibliographic data for this study are extracted from Thomson Reuters’ Web of Science (WoS) database. The research focus is specifically on the peer-reviewed articles published in the journals listed under the ‘Engineering and Technology’ discipline by the U.S. National Science Foundation’s (NSF) Science and Engineering (S&E) classification scheme. The NSF classification categorizes engineering into 13 subfields, namely *Aerospace Technology*, *Chemical Engineering*, *Civil Engineering*, *Computers*, *Electrical Engineering & Electronics*, *General Engineering*, *Industrial Engineering*, *Materials Science*, *Mechanical Engineering*, *Metals & Metallurgy*, *Miscellaneous Engineering & Technology*, *Nuclear Technology*, and *Operations Research*. Contrary to the WoS disciplinary classification, the NSF’s classification assigns each journal to only one discipline and one specialty, which avoids multiple counts of articles published in multidisciplinary journals.

Full given names of the authors—which are essential to assigning gender to authors—are provided in the WoS from the year 2008 onwards. Gender of authors was assigned to each author using the universal and country-specific existing name and gender databases, among which are U.S. Census, WikiName, Wikipedia, France and Quebec lists, and country-specific lists. Gender is assigned to the authors using first the U.S. Census and then using country-specific lists for the authors affiliated to that specific country (with the use of affiliation of the authors indexed in the WoS). More details on the method can be found in [6]. A total of 679,338 engineering articles were extracted from the Web of Science database, along with their 974,837 authorships. Authors’ affiliations were categorized into academic, governmental, and industrial sectors using the keyword filters listed in S1 Table.

### Bibliometrics

The quantitative analysis of this research is grounded on bibliometric indicators of scientific production. Bibliometrics is a quantitative method used to measure scientific activity through the analysis of the scientific publications and of their metadata. To evaluate the scientific contribution and impact of authors of each gender, this study uses the number of scientific publications as an indicator of scientific output of engineering researchers, the normalized citation count to address scientific quality and impact, and the field-normalized journal Impact Factor (IF) as a journal impact indicator, to take into account different citation practices across subfields of engineering. The proportion of scientific output of each gender is measured as a fractional count of articles, assigning each author 1/*x* count of authorship where *x* represents the number of co-authors of an article to which a gender is attributed. In this study, authorship value is attributed to these gendered fractions and is aggregated at discipline and sector level.

Citation measures are calculated as the average yearly number of citations received by an article (from its year of publication to end of the year 2014) divided by the average yearly number of citations to all publications from the same year, in the same subject area and of the same document type [53,54]. The normalized journal Impact Factor, as a measure of journal quality and impact, is calculated in a similar manner, with the Impact Factor of the journal associated to each paper it published. Note that in this research, the scientific and journal impact of the publications written by female/male engineers were analyzed when they had what is typically considered as the most important contribution to a paper [55]–i.e., when they were listed as the first author.

The cross-gender citation and journal impact comparisons discussed in this study are based on significance (sig.) tests (statistical t-tests), which assume that observations are normally distributed. However, since citation and Impact Factor data follow a positively skewed distribution and are not normally distributed, bootstrapping techniques are applied to calculate a 95% confidence interval (CI) for the means of scientific impact measures of each gender and difference between the means. Bootstrapping resamples and replaces observations, reducing the effect of outliers and other data problems. T-tests (sig. level: 0.05) are therefore validated with 1000 bootstrap samples.

### Co-authorship network analysis

To study the collaboration patterns among female and male engineering researchers, this paper analyzes the co-authorship networks in each of the engineering specialties, using *Gephi* [56], a social network analysis (SNA) tool which enables visualization of large networks and calculation of network metrics. In a co-authorship network, each node represents an author affiliated with a specific institution and two nodes are linked together if two researchers co-author a paper in the same engineering subfield. It should be noted that authors with more than one affiliation are considered as more than one node, since their affiliation with different institutions may bring out different authorship characteristics. Each node (author) is characterized by gender, affiliated sector, and productivity (the full count of papers (co)authored by a researcher). For each link (edge), an attribute is defined based on whether the collaboration link is between: two female authors (FF), or a female author and a male author (FM), or two male researchers (MM). The weight of each link accounts for the number of times two authors have collaborated over the period 2008–2013.

To assess the role of individual scientists (actors) in the scientific network, this article deploys some of the main network metrics that can be applied to disconnected networks, namely degree centrality and clustering coefficient [57]. Degree centrality measures the number of distinct links a node has to other nodes or in other words the number of direct neighbors. In a co-authorship network, high degree centrality individuals are usually interpreted as the most collaborative, important, and popular researchers who have an advantaged position in the network to receive, influence and spread knowledge [58]. However, some authors might have high degree centrality in the network due to their collaboration with many authors in only one paper, rather than their collaboration with other authors in many papers [59].

The degree centrality measure varies over a wide range and follows a positively skewed distribution. Outlier nodes are detected using the box plot method and are excluded in the trend and collaboration pattern analysis. The boxplot analysis is largely used to detect outliers and is most suitable for symmetric and skewed quantitative data. Since the distribution of authors’ degree centrality is positively skewed, the most extreme values to be considered as outliers are values higher than Q3+1.5(IQR) (i.e., in this study 14.5), where Q3 is the third quartile (i.e., in this study 7) and interquartile range (IQR) (i.e., in this study 5) is the difference between third and first quartile. As the share of authors (nodes) who are outliers is very low and they represent only 7% of the authors’ population, these values (i.e., nodes with degree centrality of 15 or higher) are excluded to avoid any bias toward the extreme collaboration patterns of these researchers.

The clustering coefficient of a node is defined as the ratio of the existing links among its nearest neighbors to all their possible connection. This corresponds to how well-connected the neighbors of a node are to each other. Most of the neighbors of a node with high clustering coefficient can collaborate with one another even if the node is removed from the network [58]. In our co-authorship network, a clustering coefficient of an author is used to represent a proxy for the impact of removing this author from the network, based on which the higher clustering coefficient of an author is, the lower his/her impact is in the network.

For the network visualization, this paper applies Gephi’s *Force-Atlas 2* algorithm in which nodes repel each other and are attracted to the centre. In this layout, nodes become spatially closer if they are linked together and the heavier the weight of the edge, the closer the nodes are.

The analyses presented in this research are grounded on cross-gender and cross-sector analyses of the engineering publications, mapping women engineers’ position in different sectors of the engineering subfields. Note that general and miscellaneous engineering subfields are excluded from the cross-disciplinary analysis, as those include articles on a very comprehensive and interdisciplinary topics, usually written by engineers specialized in other specified engineering specialties.

The various specialties do not have the same level of research activity. As Table 1 reveals, materials science accounts for the largest share of engineering papers (26.04%), followed by electrical engineering (22.4%) and by computers (14.29%). From here onward in this study, engineering specialties are listed and ordered by their share of papers.

**Table 1:** Number and share of papers by engineering specialty.

Engineering specialties	Number of papers	Share of papers
Materials Science	176,885	26.04%
Electrical Engineering & Electronics	152,199	22.40%
Computers	97,068	14.29%
Chemical Engineering	66,368	9.77%
Mechanical Engineering	49,705	7.32%
Metals & Metallurgy	36,433	5.36%
Miscellaneous Engineering & Technology	34,097	5.02%
Civil Engineering	24,318	3.58%
Nuclear Technology	13,769	2.03%
Industrial Engineering	9,440	1.39%
Aerospace Technology	8,384	1.23%
Operations Research	7,278	1.07%
General Engineering	3,394	0.50%
**All engineering**	**679,338**	**100%**

**Fig 1:**
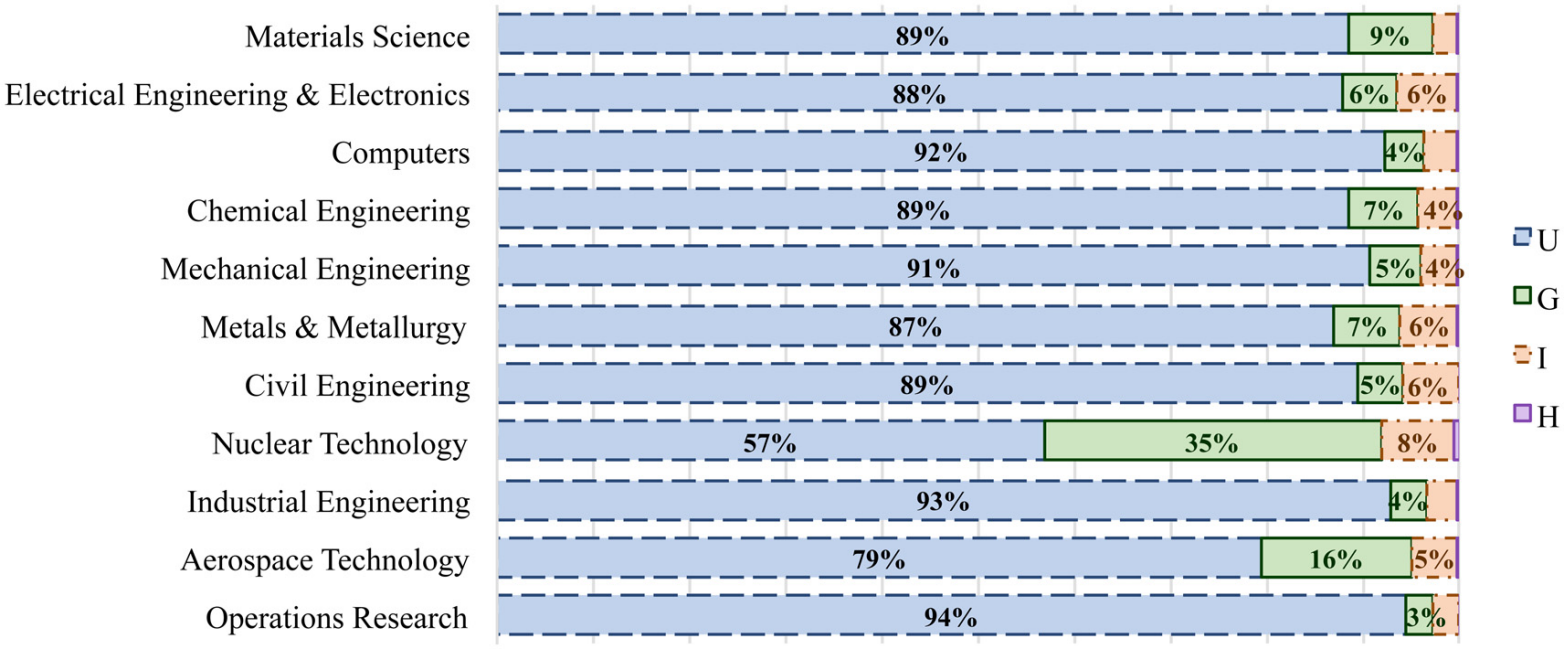
Share of different sectors (universities (U), governmental institutions (G), industry (I) and hospitals (H)) in authoring papers across various disciplines.

Universities hold large shares in authoring papers in all the engineering specialties, except in nuclear technology where the role of governmental agencies and companies is quite important. Government have also a strong share in research in aerospace technology and materials science (16% and 9%, respectively), and electrical engineering, metals & metallurgy and civil engineering obtain the highest share of industry involvement (Fig 1).

## Results

### Cross-disciplinary comparisons

**Fig 2:**
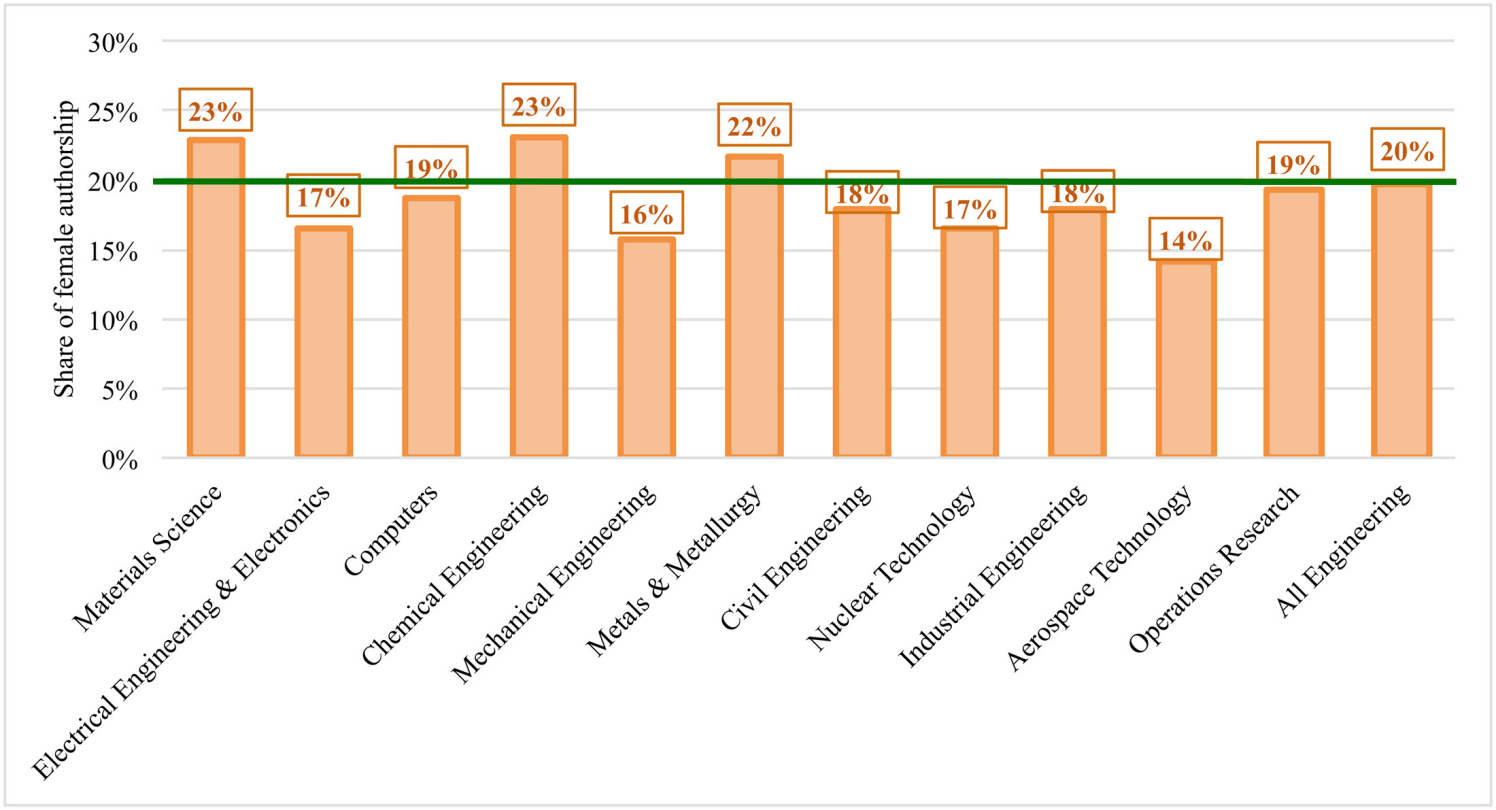
Share of female authorship in engineering specialties.

The production of scientific articles in engineering is male-dominated: the total share of women’s authorship is 20% (the green line in Fig 2), which is 10% lower than the overall share of women authorships across all scientific domains (~30%) [6]. First-authored female papers appeared in journals with significantly higher field-normalized Impact Factors than their male colleagues, but received significantly fewer citations (Table 2). First-authored female papers also obtained significantly lower citation rates even in materials and metals & metallurgy subfields where they account for a larger share of the scientific output (23% and 22%, respectively). In most male-dominated engineering specialties (i.e., aerospace technology, mechanical engineering, electrical engineering, and nuclear technology), first-authored female papers were published in significantly higher ranked journals and typically obtained a similar (not significantly different) number of citations to those first-authored by men. Civil engineering was the only engineering specialty where papers first-authored by women received significantly higher number of citations (Table 2).

**Table 2:** 95% Cls for average citation and journal impact of the papers first-authored by female and male engineers and significance tests for their differences across engineering specialties (* means that the difference is statistically significant).

Engineering specialties	Measure	Mean (CI) -Female	Mean (CI)-Male	Mean Difference (Female-Male)	Sig. (two—tailed)
**Materials Science**	**Citation**	1.14 (1.11–1.17)	1.23 (1.21–1.24)	-0.09	0.001*
	**IF**	1.14 (1.12–1.15)	1.18 (1.17–1.19)	-0.04	0.001*
**Electrical Engineering & Electronics**	**Citation**	1.15 (1.12–1.17)	1.13 (1.12–1.14)	0.02	0.233
	**IF**	1.09 (1.08–1.10)	1.05 (1.05–1.05)	0.04	0.001*
**Computers**	**Citation**	0.99 (0.96–1.02)	1.12 (1.10–1.14)	-0.13	0.001*
	**IF**	1.05 (1.04–1.06)	1.03 (1.02–1.04)	0.02	0.012*
**Chemical Engineering**	**Citation**	1.16 (1.13–1.20)	1.18 (1.16–1.20)	-0.02	0.432
	**IF**	1.13 (1.12–1.15)	1.14 (1.13–1.14)	0.00	0.599
**Mechanical Engineering**	**Citation**	1.11 (1.05–1.16)	1.06 (1.04–1.08)	0.05	0.104
	**IF**	1.08 (1.06–1.11)	1.01 (1.00–1.02)	0.07	0.001*
**Metals & Metallurgy**	**Citation**	1.11 (1.07–1.15)	1.17 (1.14–1.20)	-0.06	0.019*
	**IF**	1.15 (1.13–1.17)	1.13 (1.12–1.14)	0.02	0.131
**Civil Engineering**	**Citation**	1.16 (1.11–1.21)	1.08 (1.05–1.10)	0.08	0.007*
	**IF**	1.04 (1.02–1.06)	0.99 (0.98–1.00)	0.05	0.001*
**Nuclear Technology**	**Citation**	1.03 (0.97–1.10)	0.99 (0.95–1.04)	0.04	0.335
	**IF**	1.05 (1.03–1.06)	1.01 (1.00–1.02)	0.04	0.002*
**Industrial Engineering**	**Citation**	1.02 (0.92–1.12)	1.06 (1.02–1.10)	-0.04	0.490
	**IF**	0.99 (0.97–1.02)	1.05 (1.03–1.06)	-0.05	0.001*
**Aerospace Technology**	**Citation**	1.12 (1.03–1.22)	1.14 (1.10–1.19)	-0.02	0.654
	**IF**	1.10 (1.07–1.13)	1.07 (1.06–1.08)	0.03	0.039*
**Operations Research**	**Citation**	1.14 (1.05–1.24)	1.11 (1.06–1.15)	0.03	0.573
	**IF**	1.03 (1.00–1.06)	1.06 (1.04–1.07)	-0.03	0.114
**All Engineering**	**Citation**	1.11 (1.10–1.13)	1.14 (1.14–1.15)	-0.03	0.001*
	**IF**	1.10 (1.09–1.11)	1.08 (1.08–1.09)	0.02	0.001*

### Cross-sector comparison

**Fig 3:**
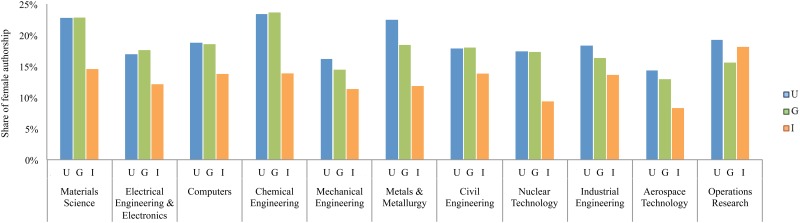
Share of female authorships by type of sector across different disciplines.

There is a higher share of female authorship in universities and governmental institutes as compared to the industrial sector across all engineering specialties except the operations research subfield where the share of female authorship in industry is almost as high as academia (Fig 3). This might be due to the fact that research in operations research is quite interdisciplinary, spanning into the field of management.

**Fig 4:**
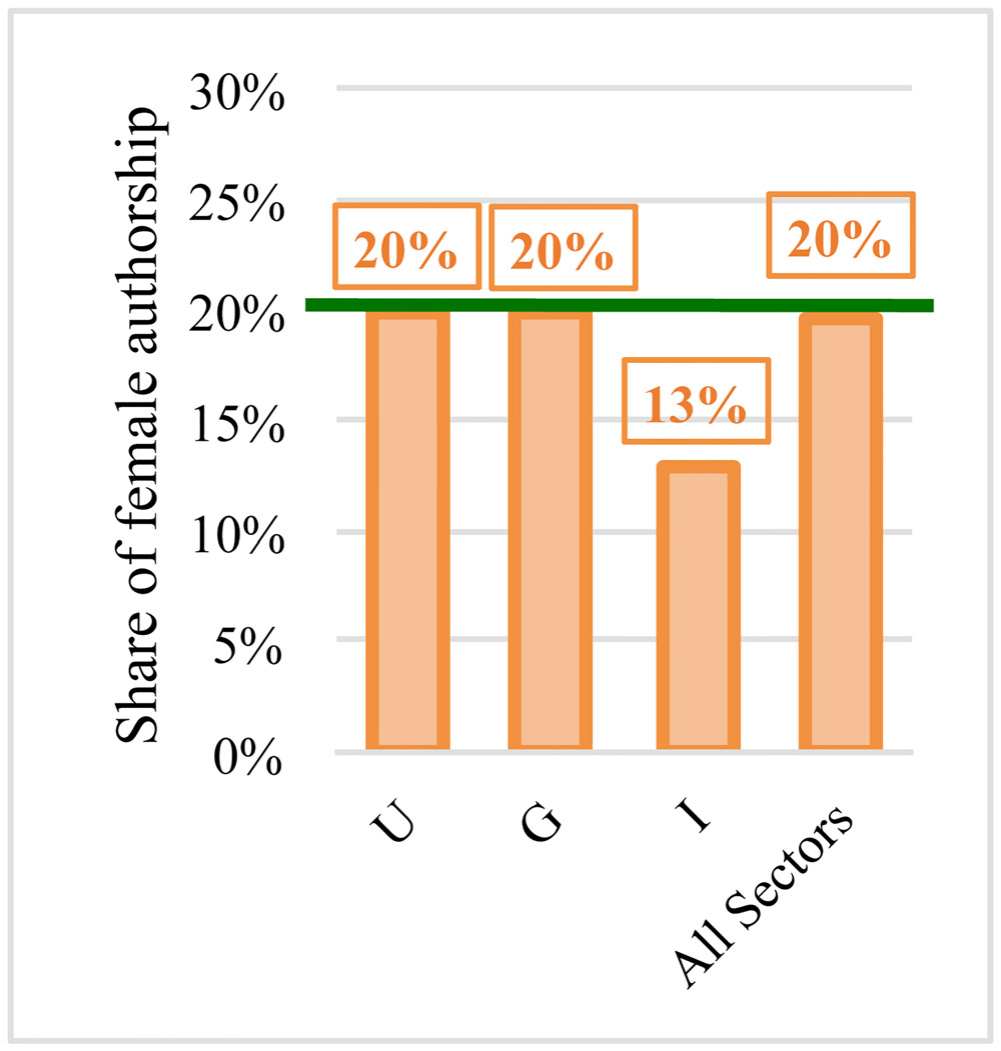
Share of female authorship in different sectors.

The share of female authorship is only 13% at industry level (Fig 4). However, female engineers affiliated with companies published in journals with significantly higher Impact Factor and the number of citations received by their papers did not differ significantly compared to that of their male counterparts, despite the fact that the scientific impact of research is, in general, lower in industry than in academe and the government (Table 3).

**Table 3:** 95% Cls for average citation and journal impact of the papers first-authored by female and male engineers and significance tests for their differences across various sectors (* means that the difference is statistically significant).

Sector	Measure	Mean (CI) -Female	Mean (CI)-Male	Mean Difference (Female-Male)	Sig. (two—tailed)
**University**	**Citation**	1.13 (1.12–1.15)	1.17 (1.17–1.18)	-0.04	0.001*
	**IF**	1.10 (1.10–1.11)	1.09 (1.09–1.10)	0.01	0.003*
**Government**	**Citation**	1.18 (1.13–1.24)	1.24 (1.21–1.27)	-0.05	0.084
	**IF**	1.23 (1.21–1.26)	1.22 (1.20–1.23)	0.01	0.329
**Industry**	**Citation**	0.85 (0.79–0.91)	0.85 (0.82–0.88)	0.00	0.946
	**IF**	0.95 (0.92–0.98)	0.90 (0.89–0.91)	0.05	0.005*
**All sectors**	**Citation**	1.11 (1.10–1.13)	1.14 (1.14–1.15)	-0.03	0.001*
	**IF**	1.10 (1.09–1.11)	1.08 (1.08–1.09)	0.02	0.001*

### Network analysis

Table 4 provides the size and properties of the co-authorship networks of each subfield of engineering. On average, materials and nuclear engineers contribute to papers that have high number of authors and are more closely linked with each other (higher average degree centrality) than researchers of the other subfields and, hence, are more collaborative. The higher clustering coefficient in these subfields also suggests that those engineers are involved in more cohesive and interconnected scientific communities. However, there is a significant difference between the two networks. Materials scientists are very productive—and thus their collaborations span across a large number of papers—whereas researchers in nuclear technology are less productive than researchers in most other areas of engineering, but contribute to lower number of papers that have a very high number of authors (Table 4).

**Table 4:** Network size and network properties across engineering disciplines.

	Nodes	Edges	Average degree	Average clustering coefficient	Average author per paper	Average productivity
**Materials science**	327,302	1,178,613	7.20	0.76	3.88	2.41
**Electrical Engineering & Electronics**	241,981	657,571	5.44	0.70	3.04	2.29
**Computers**	157,155	342,747	4.36	0.66	2.84	2.07
**Chemical Engineering**	122,358	289,668	4.74	0.74	3.26	2.21
**Mechanical Engineering**	77,890	130,063	3.34	0.64	2.62	2.28
**Metals & Metallurgy**	67,160	156,860	4.67	0.74	3.28	2.51
**Civil Engineering**	38,507	58,852	3.06	0.62	2.53	1.95
**Nuclear Technology**	29,950	105,959	7.08	0.77	3.73	1.88
**Industrial Engineering**	17,201	24,407	2.84	0.62	2.53	1.99
**Aerospace Technology**	15,871	29,574	3.73	0.68	2.73	1.81
**Operations Research**	11,897	15,757	2.65	0.57	2.44	2.04
**All Engineering**	974,837	2,998,178	6.15	0.69	3.22	2.24

**Fig 5:**
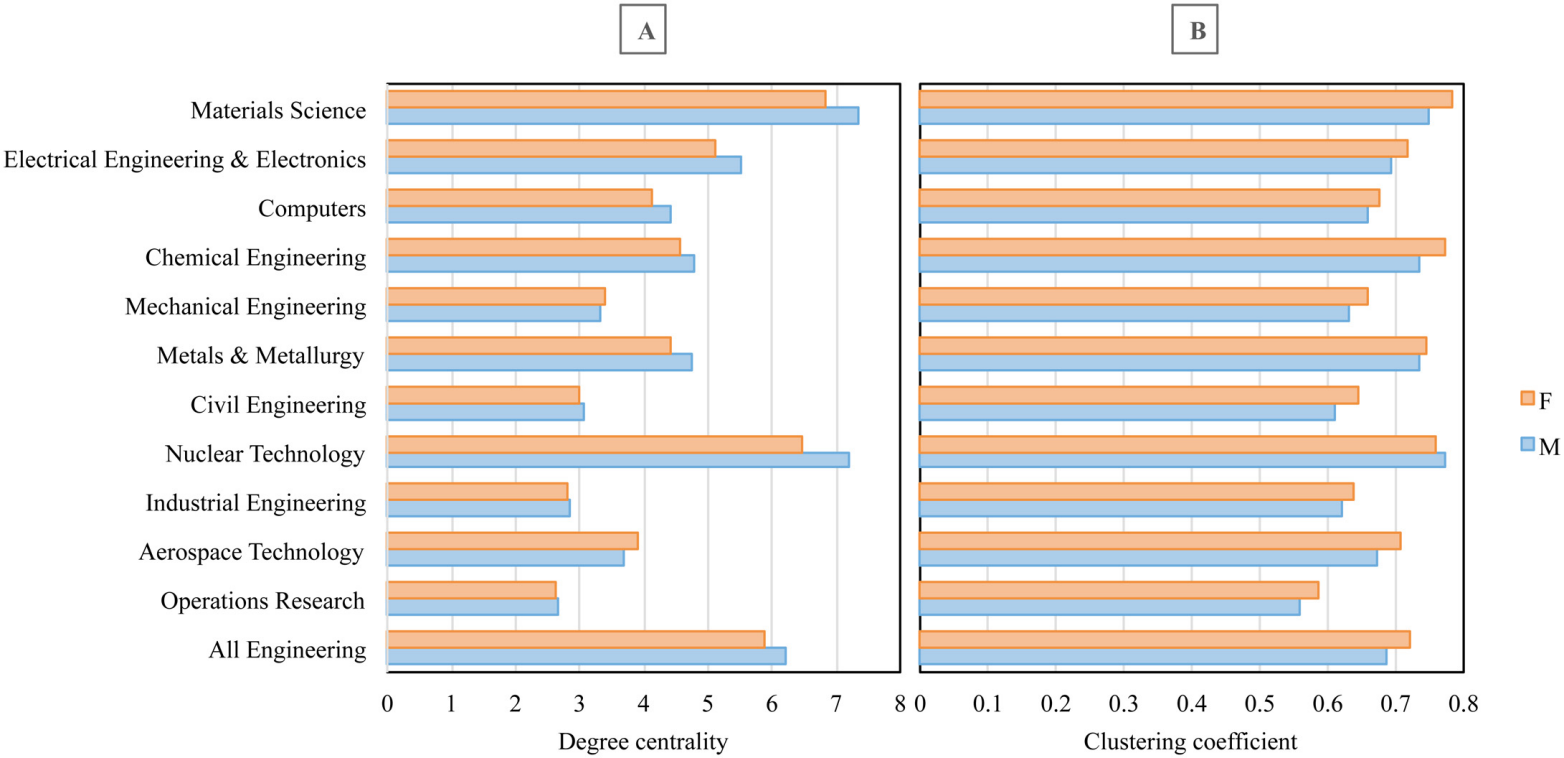
Degree centrality (A) and clustering coefficient (B) of female and male researchers across engineering specialties.

In engineering collaboration networks, women occupy less central positions compared to their male colleagues. This is observed across all specialties of engineering except from aerospace and mechanical engineering (Fig 5A). Despite the fact that papers published in these disciplines contain the lowest share of female authorships, female aerospace and mechanical engineers are shown, on average, to be more influential and have broader collaborative practices than their male peers. The average clustering coefficient is higher for women across almost all the disciplines—except in nuclear technology—which signifies that the people with whom female engineers collaborate are well-connected and can still communicate with each other even when women are removed from the network (Fig 5B).

**Fig 6:**
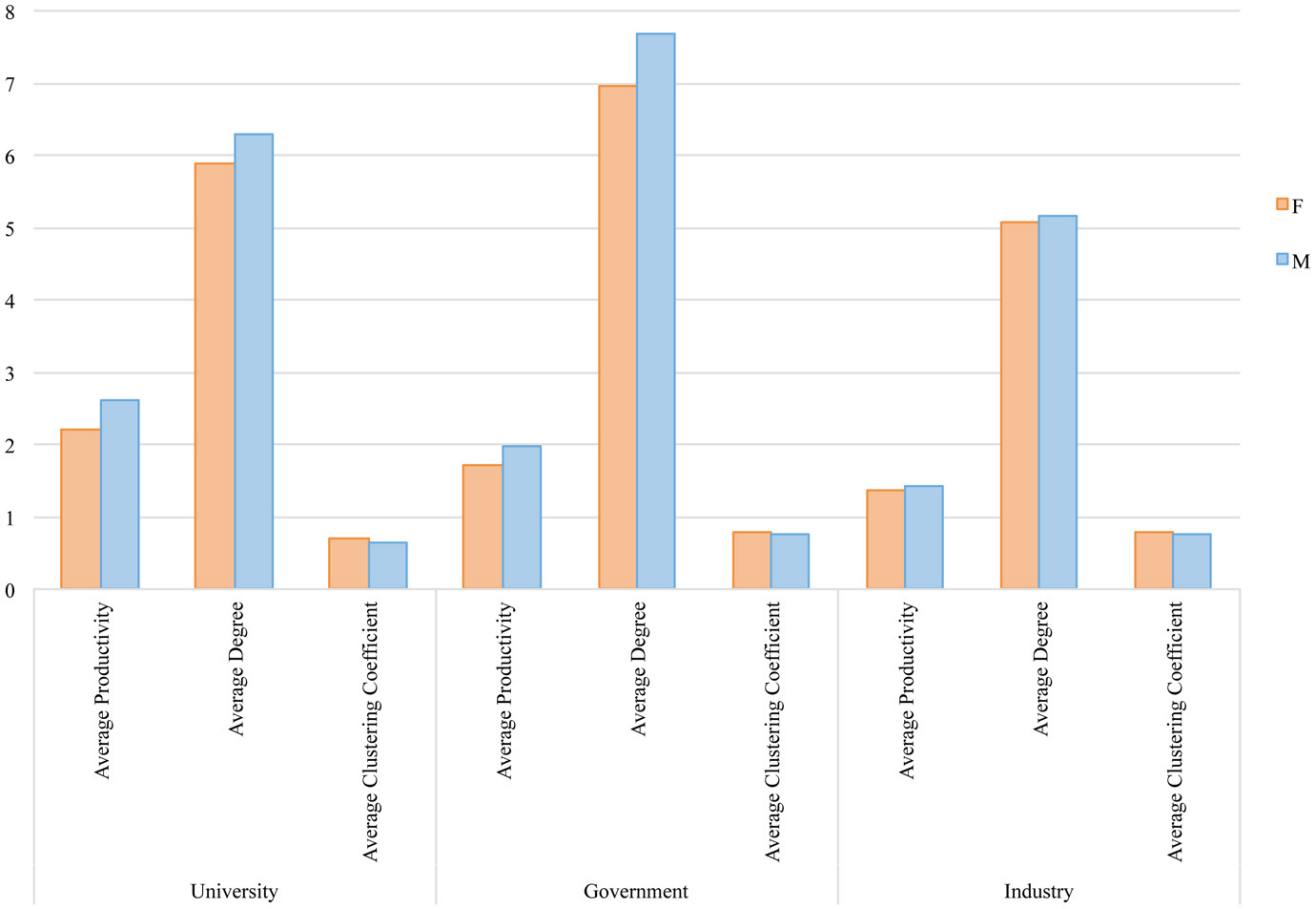
Productivity, degree centrality and clustering coefficient of female and male researchers across different sectors.

Researchers affiliated to universities tend to be the most productive, while researchers from governmental agencies are more central and have higher number of co-authorship collaborations. This is due to the fact that the average number of authors per paper is higher at the governmental level (~3.7) than at the academic and industrial levels (~3.2). Female researchers affiliated to industry have been shown to be as central and productive in the network as their male colleagues (Fig 6). Therefore, in addition to contributing to research that has a equal or higher impact, women engineers are equally or more productive, collaborative, and central than their male counterparts in the sectors and fields where they are least represented (share of female authorship is the lowest). This agrees with the conclusion of Dryburgh [49] in the context of engineering workplace culture that in order to compensate for being a woman, women engineers need to be significantly more competent in their work than their male counterparts.

### Types of collaborations

**Fig 7:**
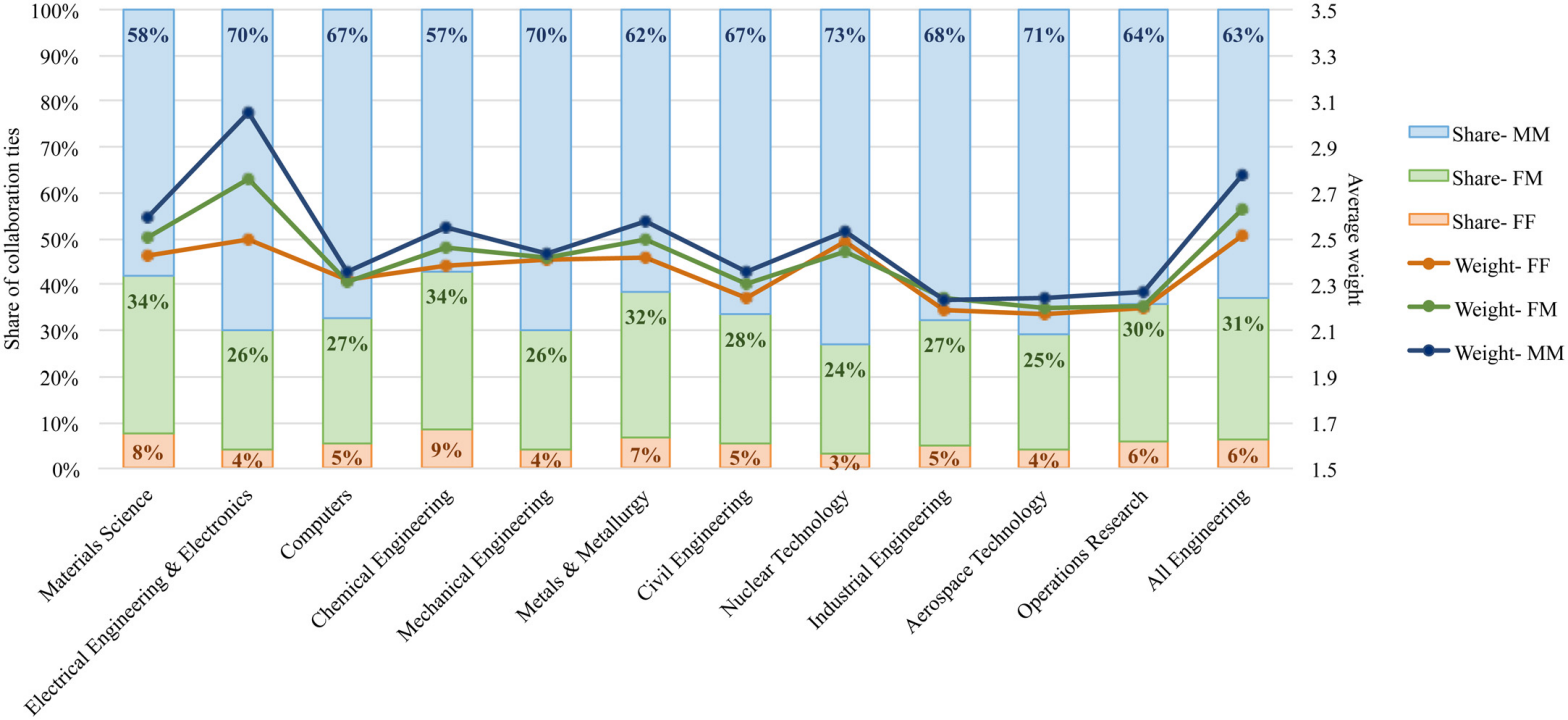
Share and average weight of FF, FM, and MM collaborations across engineering specialties.

In attempt to understand how individuals of each gender collaborate with one another, links in the networks are attributed to three collaboration types: (1) a link between a female engineer with another female engineer (FF collaboration), (2) a link between a female engineer with a male engineer (FM collaboration), and (3) a link between a male engineer with another male engineer (MM collaboration). MM collaborations account for the lion’s share of co-authorship collaborations, while FF collaborations form less than 7% of total collaborations among engineers (Fig 7). These results are expected, given that the field of engineering is largely male-dominant and the number of female engineers is relatively low, hence FF collaboration are less likely to form. At the level of engineering specialties, different results are obtained (Fig 7). The share of FF ties is the highest in specialties where share of female authorship is the highest (i.e., materials, chemical engineering) and is the lowest among the most male-dominated ones (i.e., aerospace, mechanical, and nuclear engineering). Although low in number, FF collaborations outweigh FM collaboration ties in nuclear technology. This shows that women nuclear engineers formed stronger collaboration ties with their female counterparts than with their male peers or, in other words, they *repeated* their collaboration on authoring papers more with women despite of *having* higher number of collaborations with men. The weights of collaborations for co-authorship relations are similar for all three collaboration types in mechanical engineering and computers subfields, showing that FF collaborations are as strong as FM and MM collaborations. It can be interpreted that researchers in those subfields repeated their collaborations with women as much as with men.

**Fig 8:**
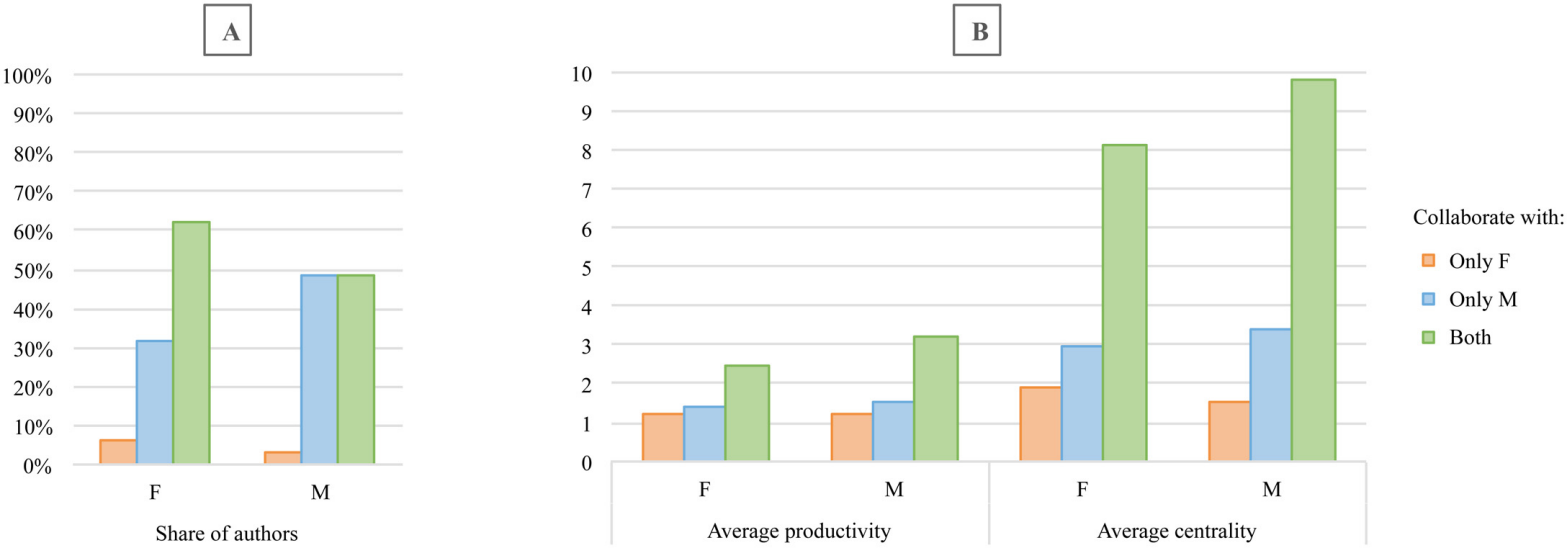
Share (A), average productivity and degree centrality (B) of researchers of each gender who collaborated only with females, only with males, and with researchers of both gender.

Scrutinizing the collaboration types of each gender, the analyses reveal that less than 10% of authors of each gender have collaborated only with female engineers. On the other hand, 38% of female engineers and around 50% of the male engineers collaborate exclusively with male engineers and do not have any female collaborators. However, it is shown that researchers with a mix-gender collaboration team are more central to the network and are more productive (Fig 8).

### Collaboration patterns

To map the co-authorship collaboration patterns of researchers of each gender, this study classifies engineers based on their gender and total number of collaborators (i.e., degree centrality) and examines differences among female and male engineer’s collaboration teams, in terms of gender disparities inside the team and the central position of researchers with whom they are collaborating. For this purpose, nodes with outlier degree centrality value are excluded and the analysis is based on female and male engineers with degree centrality values of 1 to 14.

**Fig 9:**
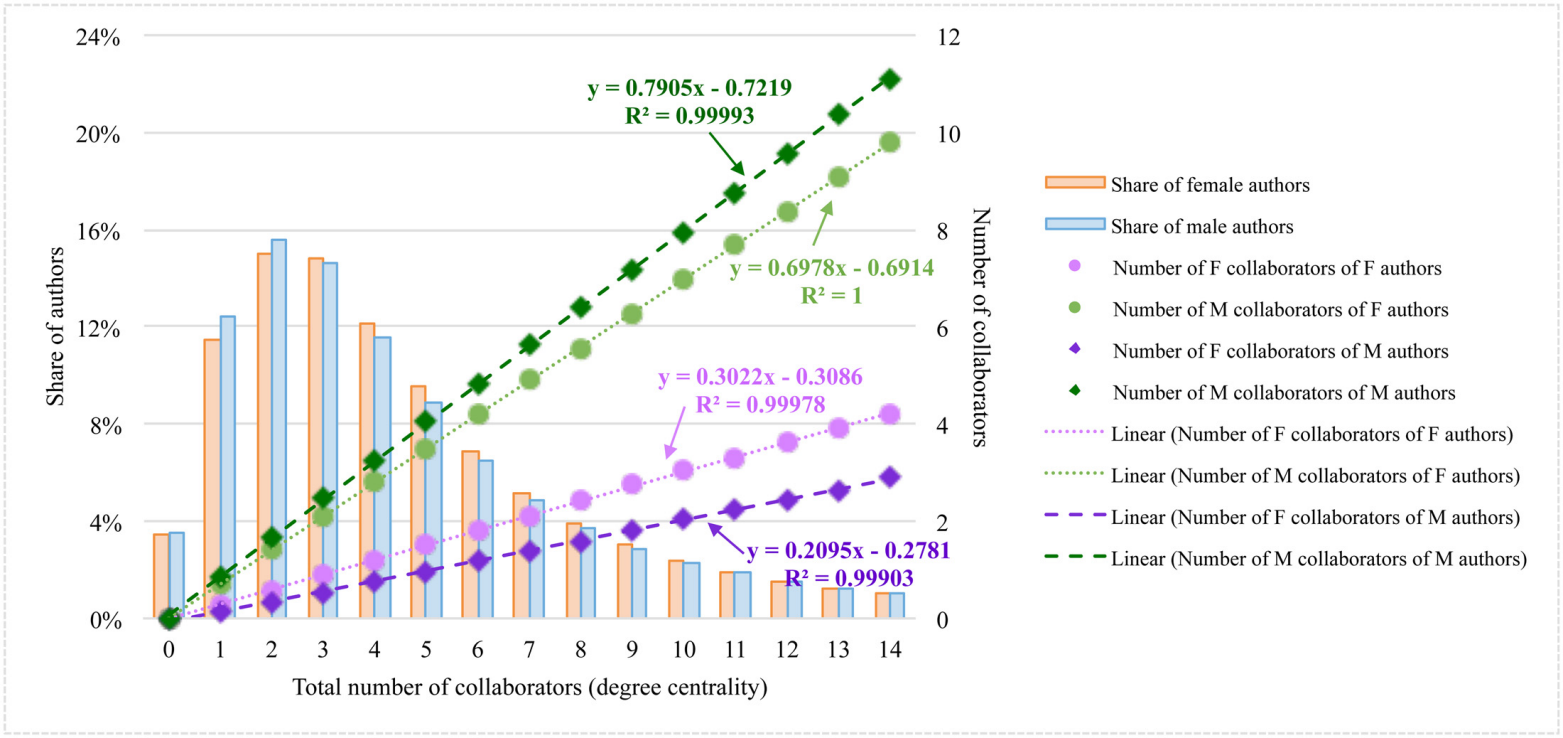
Degree centrality distribution of female and male authors (left axis), and average number of female and male collaborators of female and male engineers (right axis).

Engineers of both genders tend to co-author mostly with 2 or 3 people (or, in other words, they frequently are engaged in the collaboration team size of 3 or 4 engineers), but regardless of their gender, they tend to have more male collaborators than females—this is inevitable in a male-dominant system. However, within the same team size (i.e., the same degree centrality value), the gender gap is higher among male engineers’ direct collaborators when compared to women’s. The number of male and female collaborators of a female engineer follows a trend line different than that of a male engineer as the number of collaborators (degree centrality) increases. The gap in terms of female and male collaborators is lower for female engineers than for male engineers, which suggests that female engineers’ teams are more gender-balanced. The differences in the slopes of trend lines show that women include male engineers 2.3 times more than female engineers in their collaboration teams—female and male engineers form, respectively, 30% and 70% population of a female engineer’s direct co-authors—whereas male engineers choose men as their collaborators 4 times more often than women (Fig 9).

**Fig 10:**
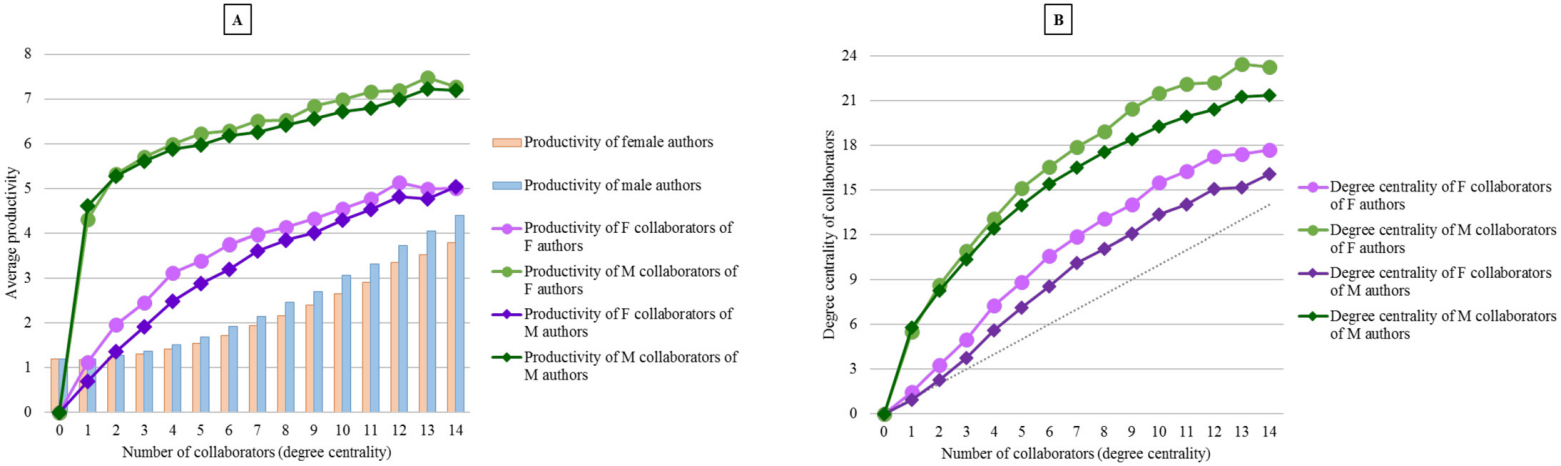
Productivity (A) and degree centrality (B) of female and male collaborators of female and male engineers.

Fig 10 demonstrates that direct collaborators of an author are more central (or productive) if the author is himself more central (or productive), which suggests that assortative mixing [60] might be present in the engineering co-authorship network. For engineers, productivity increases as their co-authorship team size increases (Fig 10A). Regardless of the gender and degree centrality level of an author, his/her male collaborators are on average more central and productive than their female collaborators. However, collaboration patterns of female engineers are slightly different from that of their male peers with the same number of collaborators: women engineers build their co-authorship team with authors who are on average more central and productive than men engineers’ collaborators. In other words, women need to include more central and productive researchers in their collaboration team in order to attain the same central position as that of men (Fig 10B).

### Network visualizations

**Fig 11:**
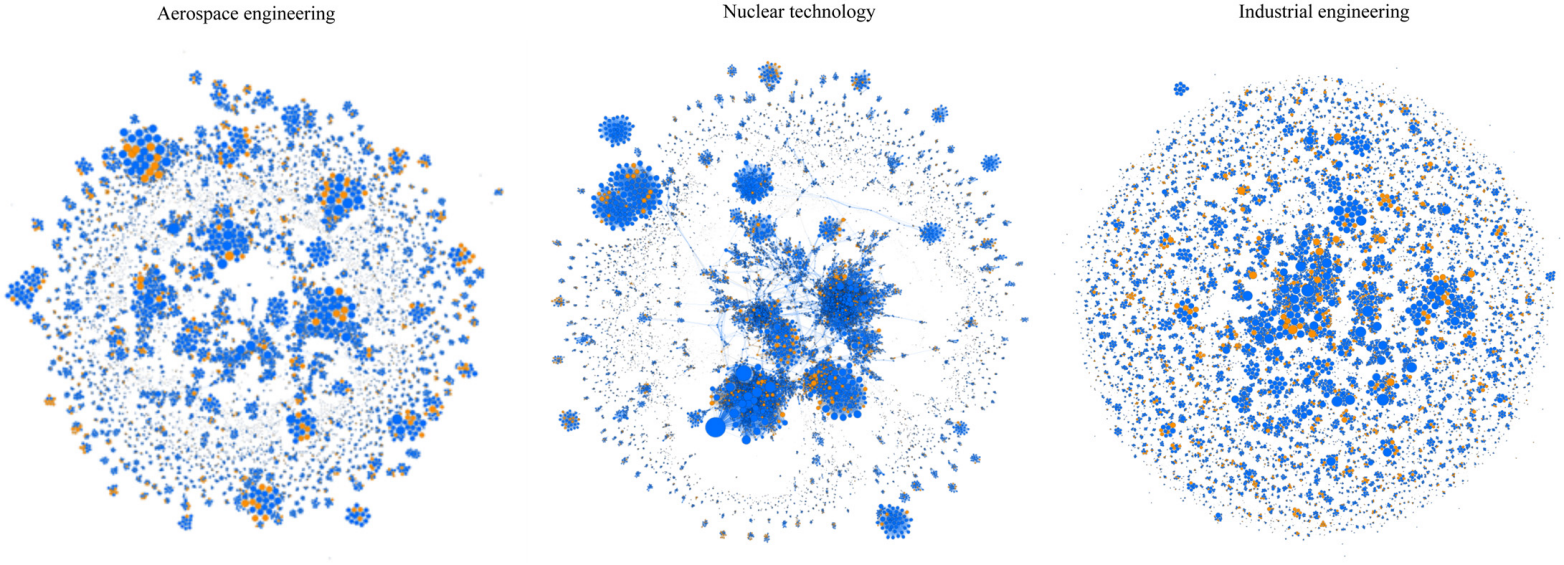
Visual presentation of co-authorship network in aerospace engineering (left), nuclear technology (middle), and industrial engineering (right).

Fig 11 provides a visualization of the co-authorship networks of aerospace, nuclear, and industrial engineers, respectively. Since the visualization boundary is limited in Gephi, the display of very large-scale networks is extremely dense (known as hairballs) and is not readable. The three specialties of aerospace, nuclear, and industrial engineering are selected because of their proper size and their distinct characteristics.

Blue nodes represent male authors and orange females. The size of each node is based on its degree centrality. Clusters in a co-authorship network represent authors who work closely because of either their research and expertise on the same (or complementary) subject or the geographical proximity of their affiliations [61].

These visual representations offer new insights on the position of female researchers in a network. The figure shows that women engineers occupy highly central positions in cases where the networks comprise several smaller “disconnected clusters” (e.g., aerospace engineering), while women are less central in networks with few well-connected clusters (e.g., nuclear technology). They are equally central in a network composed of both disconnected and connected clusters (e.g., industrial engineering).

## Discussion

The engineering scientific system is highly male-dominated: women account for only 20% of total authorship. Women engineers publish their papers in journals with higher citations rates, while their work receives fewer citations from the engineering community. Similar results were obtained by Larivière [62] at the level of all disciplines combined. This finding can be interpreted within the framework of the “Matilda effect” [63] by which women’s publications receive less recognition than what is expected (in this case, expected from the journal in which their discoveries were published).

Despite the gender differences, women are equally or more influential and prominent than their male peers across fields and sector where they are *least* represented—i.e., those fields and sectors in which their authorship share is lowest (fields: aerospace and mechanical engineering; sector: industry): they published in higher ranked journals and their papers received as many citations as their male counterparts. Co-authorship network analysis also reveals that women occupy more central and important positions, again, across most male-dominated fields and sector. This may suggest a strong selection effect: unless being extremely qualified and accomplished, women tend to leave the field.

In a knowledge network, researchers with high degree centrality are associated with prominent positions, in which they might have better access to information and resources and hence might be able to influence the scientific system [64]. In engineering, in order to have the same degree centrality as their male peers, women engineers tend to include more highly central researchers in their collaboration teams than men do. In a male-dominated environment, women engineers might need to make a considerable effort to collaborate with prominent engineers as they tend to collaborate mostly with men. Hence, women might need to work harder to fill the same position and access the same resources as their male colleagues. These findings conform with the discussions on the masculine culture of engineering workplace, where women engineers are required to compensate for their gender by being exceptionally competent [49] and putting extra effort [52] into their work beyond what is required from men in a same or similar position.

Engineers, in general, form and repeat their co-authorship collaboration more with men. However, authors in mechanical engineering and computers subfields *repeat* their collaborations irrespective of the gender of their collaborators. Note that this *does not* reflect gender blindness in their *selection* of co-authors; it simply means that mechanical and computer engineers collaborate with women on as many articles as they do with men.

On the selection of female co-authors, network visualization suggests that the centrality of women might be dependent on the size of the cluster where they are located. Women engineers are more central in networks composed of several tight “disconnected clusters”. This is explained by noting that when a cluster is disconnected and small, it represents a community of few authors involved in a very specific research topic [65]. Due to the high level of expertise, the number of researchers collaborating on a specific research problem is often very limited. Therefore engineers in these clusters (communities) might have lower selection choice of collaborators and women, hence, may appear as an important channel of scholarly communication and activity. This implies that women’s propensity to specialize less than men [22] might play out as a barrier to occupy central positions in their scientific network.

Along these lines, collaboration patterns of men have shown to be even less gender balanced, with about 50% of male researchers being strictly engaged in collaborations with other male colleagues—despite the higher centrality and productivity of researchers with more gender-balanced collaboration practices. This subset of researchers includes fewer women in collaboration teams and builds stronger connections with their male counterparts. Although women are more likely to include other women in their collaborations, these collaboration ties are weaker than their ties with men engineers and they repeat their co-authorship collaborations more with their male counterparts. Therefore, it can be said that women engineers are complying with the male-dominant engineering scientific system instead of changing its structure—i.e., Gupta’s [15] first possible response to triple burden. Men are also contributing to the reproduction of these male-dominated scientific structures, which disfavors women in the sense that engineers—regardless of their gender—collaborate and repeat collaborations predominately with men.

## Conclusion

Women’s collaboration patterns in engineering shed light on the lower gender gap among their collaborators, which combined with the right policy support might frame a scientific system that promotes higher co-authorship rate and collaboration weight with women. As researchers involved in mix-gendered collaboration teams outperform their peers involved in single-gendered teams (in terms of level of productivity and degree centrality), this study calls for policies to support engagement of women in engineering research and improve collaboration with them.

Women’s participation in engineering represents a valuable human resource in development of scientific knowledge and technology. The ‘gendered’ and ‘gendering’ aspect of STI policy structures plays a major role in creation of masculine academic and organizational cultures which acts as a barrier for women to enter, stay active, and gain recognition as a professional in science and technological fields. The results of this study can contribute to more effective policymaking, serving as a baseline from which to strengthen gender mainstreaming in STI capacity building programmes. The introduction and implementation of gender-responsive policies into existing S&T discourse help address the cultural factors that impede women from participating or advancing in engineering and gear a society towards higher knowledge capacity, and scientific and innovative excellence, upon which a nation’s competitive edge in the global economy is grounded.

## Supporting Information

S1 Table Filter keywords for institutional sectors (regular expressions). (DOCX)
