# Supplementary material for: On the Compliance of Women Engineers with a Gendered Scientific System
Source: PLoS One. 2015 Dec 30;10(12):e0145931. doi: 10.1371/journal.pone.0145931 (PMC4696668; doi:10.1371/journal.pone.0145931)
Supplement: S1 Table — (DOCX) [file pone.0145931.s001.docx]

**S1 Table. Filter keywords for institutional sectors (regular expressions)**

| University | Government | Industry | Hospital |
| --- | --- | --- | --- |
| [[:<:]]univ[[:>:]]  [[:<:]]universit.*[[:>:]]  [[:<:]]uniw.*[[:>:]]  [[:<:]]colleg.*[[:>:]]  [[:<:]]coll[[:>:]]  [[:<:]]school.*[[:>:]]  [[:<:]]scuola[[:>:]]  [[:<:]]ecole?[[:>:]]  [[:<:]]escola[[:>:]]  [[:<:]]sch[[:>:]]  [[:<:]]acad.*[[:>:]]  [[:<:]]akad.*[[:>:]] or  [[:<:]]inst.*(tech.*\|sci.*)[[:>:]]  [[:<:]]inst.*tecn.*[[:>:]]  [[:<:]]fac[[:>:]]  [[:<:]]facu.*[[:>:]]  [[:<:]]polytec.*[[:>:]]  [[:<:]]politec.*[[:>:]]  [[:<:]]mit[[:>:]]  [[:<:]]eth[[:>:]]  [[:<:]]rhein.*westfal.*[[:>:]]  [[:<:]]chalmers[[:>:]]  [[:<:]]caltech[[:>:]]  [[:<:]]ucla?[[:>:]]  [[:<:]]virginia-tech[[:>:]]  ^tu-.* | [[:<:]]nationale?[[:>:]]  [[:<:]]natl.*[[:>:]]  [[:<:]]nation.*[[:>:]]  [[:<:]]nat[[:>:]]  [[:<:]]nacl[[:>:]]  [[:<:]]nazl[[:>:]]  [[:<:]]minist[[:>:]]  [[:<:]]army.*[[:>:]]  [:<:]]navy[[:>:]]  [[:<:]]air-?force[[:>:]]  [[:<:]]def[[:>:]]  [[:<:]]defen.*[[:>:]]  [[:<:]]council[[:>:]]  [[:<:]]consejo[[:>:]]  [[:<:]]agcy[[:>:]]  [[:<:]]agency[[:>:]]  [[:<:]]agenc.*[[:>:]]  [[:<:]]fed[[:>:]]  [[:<:]]feder.*[[:>:]]  ^europ.*  ^state-  [[:<:]]csiro[[:>:]]  [[:<:]]nasa[[:>:]]  [[:<:]]usaf[[:>:]]  [[:<:]]nsf[[:>:]]  [[:<:]]nrc[[:>:]]  [[:<:]]cnrs[[:>:]]  [[:<:]]csic[[:>:]]  [[:<:]]cnr[[:>:]]  [[:<:]]kaeri[[:>:]]  [[:<:]]etri[[:>:]]  [[:<:]]barc[[:>:]]  [[:<:]]usn[[:>:]]  [[:<:]]csir[[:>:]]  [[:<:]]paul-scherrer-inst[[:>:]]  [[:<:]]nist[[:>:]]  [[:<:]]astar[[:>:]]  [[:<:]]cern[[:>:]]  [[:<:]]inria[[:>:]] | [[:<:]]limit.*[[:>:]]  [[:<:]]ltd[[:>:]]  [[:<:]]corp[[:>:]]  [[:<:]]corpor.*[[:>:]]  [[:<:]]compag.*[[:>:]]  [[:<:]]compan.*[[:>:]]  [[:<:]]co[[:>:]]  [[:<:]]inc[[:>:]]  [[:<:]]incor.*[[:>:]]  [[:<:]]entreprise[[:>:]]  [[:<:]]firm.?[[:>:]]  [[:<:]]consul.*[[:>:]]  [[:<:]]gmbh.*[[:>:]]  [[:<:]]plc.*[[:>:]]  [[:<:]]llc.*[[:>:]]  [:<:]]ag[[:>:]]  [[:<:]]ibm[[:>:]]  [[:<:]]ntt[[:>:]]  [[:<:]]boeing[[:>:]] | [[:<:]]hospital[[:>:]]  [[:<:]]hosp[[:>:]]  [[:<:]]hop[[:>:]]  [[:<:]]infirm.*[[:>:]]  [[:<:]]clin[[:>:]]  [[:<:]]hop[[:>:]]  [[:<:]]osped[[:>:]]  [[:<:]]szpital[[:>:]]  [[:<:]]krankenhaus[[:>:]]  [[:<:]]spital[[:>:]] |
